# Evaluating Neutrophil Gelatinase-Associated Lipocalin in Pediatric CKD: Correlations with Renal Function and Mineral Metabolism

**DOI:** 10.3390/pediatric16040094

**Published:** 2024-12-09

**Authors:** Ruxandra Maria Steflea, Emil Robert Stoicescu, Oana Aburel, Florin George Horhat, Silviu Valentin Vlad, Felix Bratosin, Andreea-Mihaela Banta, Gabriela Doros

**Affiliations:** 1 Department of Pediatrics, “Victor Babes” University of Medicine and Pharmacy, 300041 Timisoara, Romania; steflea.ruxandra@umft.ro (R.M.S.); doros.gabriela@umft.ro (G.D.); 2 “Louis Turcanu” Emergency Hospital for Children, 300011 Timisoara, Romania; oanaduicu@umft.ro; 3 Doctoral School, “Victor Babes” University of Medicine and Pharmacy, 300041 Timisoara, Romania; andreea.banta@umft.ro; 4 Department of Radiology and Medical Imaging, “Victor Babes” University of Medicine and Pharmacy Timisoara, 300041 Timisoara, Romania; stoicescu.emil@umft.ro; 5 Department of Pathophysiology, “Victor Babes” University of Medicine and Pharmacy Timisoara, 300041 Timisoara, Romania; 6 Department of Microbiology, “Victor Babes” University of Medicine and Pharmacy, 300041 Timisoara, Romania; horhat.florin@umft.ro; 7 Department of Surgery, Faculty of Medicine, University of Oradea, 410073 Oradea, Romania; 8 Department of Infectious Disease, “Victor Babes” University of Medicine and Pharmacy Timisoara, 300041 Timisoara, Romania; felix.bratosin@umft.ro

**Keywords:** pediatrics, children, pediatric disease, pediatric nephrology

## Abstract

Background: Pediatric chronic kidney disease (CKD) requires reliable biomarkers for early detection and monitoring. Neutrophil gelatinase-associated lipocalin (NGAL) has emerged as a potential marker due to its responsiveness to renal impairment and involvement in mineral metabolism. Objectives: To evaluate serum NGAL levels in pediatric CKD patients and explore correlations with estimated glomerular filtration rate (eGFR), ferritin, calcium-phosphorus (Ca*P) product, and total serum protein. Methods: A cross-sectional study included 54 pediatric CKD patients and 29 healthy controls. Laboratory assessments encompassed serum NGAL, creatinine, ferritin, calcium, phosphorus, and total serum protein. eGFR was calculated using the Schwartz formula. Pearson correlation and linear regression analyses determined associations between NGAL and other parameters. Results: NGAL levels were significantly higher in CKD patients compared to controls (median 453 ng/mL vs. 78 ng/mL, *p* < 0.001). A strong negative correlation existed between NGAL and eGFR (r = –0.81, *p* < 0.001). NGAL showed moderate positive correlations with ferritin (r = 0.56, *p* = 0.009) and CaP product (r = 0.57, *p* = 0.006) and a moderate negative correlation with total serum protein (r = –0.36, *p* = 0.225). Regression analysis confirmed NGAL as a significant predictor of eGFR, ferritin, and CaP product. Conclusions: Elevated serum NGAL levels are associated with decreased renal function and alterations in mineral metabolism in pediatric CKD patients. NGAL may serve as a valuable biomarker for assessing disease progression and guiding clinical management in this population.

## 1. Introduction

In earlier studies, several new CKD biomarkers have been assessed to complement serum creatinine and proteinuria. The ultimate objective of the studies is to identify biomarkers that can be used to anticipate the course of CKD, better schedule outpatient follow-up appointments, and facilitate transplant referrals [1].

A biomarker appropriate for chronic kidney disease monitoring should possess little biological variability to enhance the evaluation of long-term alterations. Additionally, it should not be impacted by an individual’s age, nutritional status, or any ongoing medical issue. Several characteristics must be searched to identify a good biomarker: rapid, noninvasive, and specific measurements that are well correlated with kidney tissue pathology [2].

Neutrophil gelatinase-associated lipocalin (NGAL) is a 25 kDa protein mostly released by immune cells, such as dendritic cells, macrophages, and neutrophils. Inflammation triggers an increase in its production. Urine, plasma, and biological fluids such as brain fluid and peritoneal effluent can all have their NGAL concentrations tested [3]. NGAL is produced by activated neutrophils as an innate antibacterial factor, and it seems to have more complex activities than just the bacteriostatic effect [4].

When selecting the biological fluid to use (urine or serum) to determine NGAL, we must consider the underlying disease that leads to CKD. In pediatric patients with CKD, markers like creatinine, albuminuria, or proteinuria are still part of the disease progression monitoring plan. Despite their shortcomings, their significance in clinical practice cannot be underestimated. Each biomarker has limitations; the future is to combine well-established biomarkers with new ones to assess CKD progression [5].

Under normal circumstances, NGAL is freely filtered in glomeruli and completely reabsorbed in the proximal tubule via the endocytic receptor megalin. Its function as an early marker was particularly crucial in acute kidney damage since its expression is primarily upregulated in tubules 2–4 h after acute nephrotoxic and ischemic kidney injury [6].

We selected NGAL for our pilot study because its role has already been established in CAKUT. Wasilewska et al. demonstrated its diagnostic and prognostic role in obstructive hydronephrosis. In addition to being a diagnostic biomarker, it is a noninvasive tool to predict kidney scarring in children with VUR [7,8]

The role of NGAL in all stages of CKD is being researched, including ESRD. For patients on continuous renal replacing therapy (CRRT), NGAL is associated with key indices of iron status, and plasma NGAL levels could indicate the status of iron metabolism in patients with CKD [9]. Correlations were made regarding the association between NGAL and ferritin levels, determining that there was a substantial correlation between high serum NGAL concentrations and iron-deficient anemia [10]. Additionally, connections between cholesterol and blood protein levels were found for this group of patients, confirming the involvement of NGAL in the development of malnutrition in CKD patients undergoing hemodialysis [11].

Serum NGAL concentrations are higher in patients with decreased kidney function than in healthy controls; however, there is debate over the variations in serum NGAL levels between patients with acute kidney injury (AKI) and those with CKD. One research demonstrated that individuals with AKI exhibit elevated levels of plasma NGAL in comparison to those with CKD. However, other research has produced the opposite findings [12,13].

When monitoring the progression of CKD, we also monitor for signs of secondary hyperparathyroidism (SPHT). This condition often arises as a complication in the early stages of renal insufficiency and is an adaptive response to maintain mineral balance. It is a component of CKD-MBD. A few variables contribute to the development of SHPT in CKD, which is typified by phosphate retention, hyperphosphatemia, hypocalcemia, and decreased 1,25-vitamin D levels [14].

In addition to altering skeletal muscle structure, SHPT also interferes with the metabolism of proteins and amino acids and the synthesis, transport, and use of energy. It accomplishes this by increasing the release of alanine and glutamine, encouraging muscle proteolysis, and impeding general energy metabolism [15]. Starting with this hypothesis, we identified a study in elderly patients that found respective correlations between NGAL and Ca, P, Calcium *Phosphorus product (Ca*P), and albumin, which showed that NGAL might participate in calcium and phosphorus metabolic disorders [16].

The study hypothesized that serum NGAL levels are elevated in pediatric patients with CKD and are significantly correlated with renal function and mineral metabolism parameters. Specifically, it was posited that NGAL levels negatively correlate with eGFR and total serum protein while positively correlating with ferritin and the calcium-phosphorus product (CaP). The primary objective was to evaluate serum NGAL levels in pediatric CKD patients compared to healthy controls. Secondary objectives included determining the correlations between NGAL and eGFR, ferritin, CaP product, and total serum protein, thereby assessing NGAL’s potential role as a biomarker for CKD progression and associated metabolic disturbances.

## 2. Materials and Methods

### 2.1. Study Design

This study employed a cross-sectional design. After selecting the patients from all patients with CKD admitted to our hospital in March 2023–March 2024, we collected data for the medical records. Ethical approval was obtained from the institutional review board of the “Louis Turcanu” Emergency Hospital for Children Timisoara (no. 118/25 November 2023) and the “Victor Babes” University of Medicine and Pharmacy Timisoara (no. 68/3 October 2022). Patient privacy and confidentiality were upheld throughout the trial, and all data were de-identified before analysis. Informed consent was obtained in all cases throughout the admission chart.

PICO Statement: Population (P): Pediatric patients aged 0 to 18 years diagnosed with chronic kidney disease (CKD) stages 1–5, including those undergoing continuous renal replacement therapy, admitted to “Louis Țurcanu” Emergency Hospital for Children Timisoara; Intervention (I): Measurement of serum neutrophil gelatinase-associated lipocalin (NGAL) levels and assessment of its correlation with renal function and mineral metabolism parameters; Comparison (C): Healthy pediatric controls without CKD or concurrent acute pathologies, matched for age and sex; Outcome (O): Determination of the relationship between serum NGAL levels and estimated glomerular filtration rate (eGFR), ferritin, calcium-phosphorus product, and total serum protein, to evaluate NGAL’s utility as a biomarker for CKD progression in pediatric patients.

### 2.2. Patient Population

The study enrolled pediatric patients diagnosed with chronic kidney disease (CKD) at the “Louis Turcanu” Emergency Hospital for Children in Timisoara. CKD is characterized by abnormalities in kidney structure or function persisting for at least three months, with potential implications for overall health. Inclusion in the CKD category was based on structural abnormalities identified through imaging, and the staging of CKD was guided by the KDIGO (Kidney Disease: Improving Global Outcomes) criteria, which rely on estimated glomerular filtration rate (eGFR) and albuminuria levels [17].

Inclusion criteria encompassed children aged 0 to 18 years who were admitted to the Nephrology, Peritoneal Dialysis, or Hemodialysis Departments. Exclusion criteria were applied to children without CKD, those with CKD and concurrent acute pathologies such as urinary tract infections (UTIs) or pneumonia during the evaluation period, and those from whom serum NGAL levels were not obtained concurrently with other paraclinical assessments integral to the study.

A control group consisted of 29 patients admitted during the same timeframe, selected to ensure that the underlying disease did not influence the laboratory assessments pertinent to this study. Demographic data—including age, sex, place of origin, weight, and height—were extracted from patient records. Clinical information, such as diagnosis, co-existing conditions, and medication history, was thoroughly documented.

Controls were chosen to ensure they were free from both overt and subclinical renal issues, which is crucial for establishing a valid baseline for NGAL levels. These healthy controls were matched with the CKD patients based on age and sex to further eliminate confounding variables that might skew the results. This selection was considered essential because any underlying renal dysfunction in the control group could falsely elevate NGAL levels, potentially misleading the interpretation of its role in CKD progression.

### 2.3. Laboratory Assessment

Laboratory analyses encompassed measurements of serum creatinine, total protein levels, alkaline phosphatase (ALP), total and ionized calcium levels (Ca and Ca^2+^), a lipid profile, ferritin, and neutrophil-gelatinase-associated lipocalin (NGAL). For the patient undergoing continuous renal replacement therapy (CRRT), serum samples were collected prior to the mid-week hemodialysis session. The product of calcium and phosphorus (Ca*P) was calculated using the formula: Ca*P = (4 × serum calcium) × (3.1 × serum phosphorus) [18].

Serum samples were analyzed with a Cobas Integra 400 Plus analyzer (Roche Diagnostics, CH-6343 Rotkreuz, Basel, Switzerland). Protein electrophoresis was performed using the Hydrasys 2 Electrophoresis Systems (Sebia, Norcross, GA, USA). Samples were centrifuged at 4000 rpm for 8 min, followed by standard laboratory processing protocols. Urine specimens from each patient were examined for proteinuria. The estimated glomerular filtration rate (eGFR) was calculated employing the Schwartz formula 0.413 × height/SCr, height is measured in centimeters, and SCr in mg/d [19].

### 2.4. Statistical Analysis

Statistical analyses were conducted using MedCalc^®^ Statistical Software version 22.017 (MedCalc Software Ltd., Ostend, Belgium). The collection of identification data and clinical and paraclinical parameters was systematically recorded in a secure, computerized database using Microsoft Excel version 2312 (Build 17126.20132). Descriptive statistics provided a summary of patient demographics, clinical features, and laboratory results. Measures of central tendency were calculated using medians and interquartile ranges [IQR] for data that did not conform to parametric distribution assumptions. The study employed Pearson correlation coefficients (r) and linear regression analyses to determine the strength and direction of associations between NGAL levels and estimated glomerular filtration rate. The Pearson correlation coefficient, often represented by the symbol r, is a crucial statistical metric used to evaluate the linear relationship between two variables.

## 3. Results

Among the study cohort of 54 pediatric patients diagnosed with chronic kidney disease (CKD), males constituted a slight majority at 51.85% (*n* = 14). A significant proportion, 62.86% (*n* = 17), hailed from urban areas. The median age of the participants at the time of examination was 173 months, with a range from as young as 4 months to older adolescents, underscoring the broad age spectrum of the study group. This age diversity was accompanied by a wide variation in physical metrics, with median weights and heights at 37.3 kg (interquartile range: 20.5 to 55.7 kg) and 135.33 cm (standard deviation: ±28.75 cm), respectively, reflecting stages of growth across different pediatric age groups.

**Figure 1:**
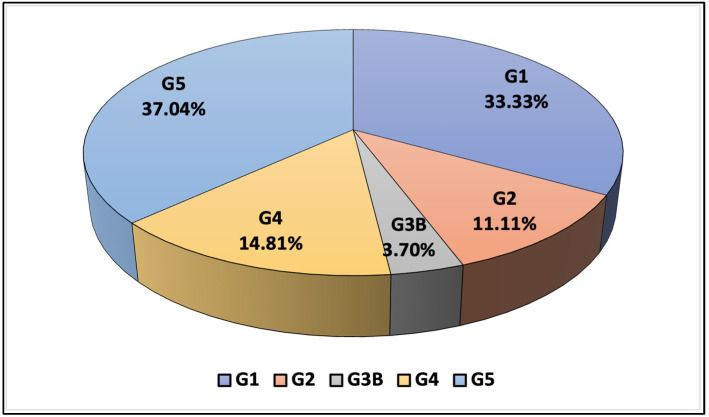
Chronic kidney disease staging according to eGFR and albuminuria.

The baseline characteristics indicate that the majority of our patients were in the mild to moderate stages of CKD, with a notable fraction, nearly 37% (*n* = 10), requiring continuous renal replacement therapy (CRRT); this subgroup included nine patients on hemodialysis and one on peritoneal dialysis, highlighting the severity of their condition. Furthermore, when CKD was classified according to albuminuria levels, a significant majority, two-thirds (66.67%) of the patients, fell into the CKD A1 category, suggesting a less severe impairment of kidney function among the majority. This demographic and clinical profile provides a foundation for analyzing the impacts of various treatments and interventions within this diverse patient population (Table 1 and Figure 1).

The causes of chronic kidney disease among the 54 pediatric patients were diverse, encompassing a range of kidney and urinary tract malformations, as detailed in the study data. Notably, neurogenic bladder was the most prevalent condition, affecting 18.52% of the cohort, and was frequently associated with vesicoureteral reflux (VUR); among these patients, four exhibited bilateral VUR, and one had undergone a nephrectomy, resulting in unilateral VUR. Renal dysplasia was identified in 11.11%, and posterior urethral valve (PUV) anomalies were found in 14.81%, which often correlated with moderate to advanced stages of CKD and included multiple malformations. Additionally, one patient with PUV also presented with Down syndrome, indicating a complex interplay of genetic and developmental factors contributing to kidney disease.

Further etiologies included collecting system anomalies such as ureteropelvic junction stenosis and ureterocele, each contributing to 7.41% and 3.7% of cases, respectively. Bladder anomalies were evident, with primary vesicoureteral reflux also observed in 3.7% of the subjects. The study highlighted other significant causes, such as chronic glomerulonephritis, with instances of IgA Nephropathy and glomerulonephritis secondary to ANCA-MPO positive vasculitis, each constituting 7.41% and 3.7% of the cases. Other less common causes included genetic conditions like nephronophthisis and systemic diseases like atypical hemolytic uremic syndrome (aHUS), underscoring the complexity and variability of CKD etiologies in pediatric patients (Table 2).

The control group consisted of children with a median age of 120 months, and gender distribution was nearly balanced, with 16 males (55.19%) and 13 females (44.81%). Residency was split between urban (62.01%) and rural (37.91%) areas. The prevalence of underlying diseases was detailed, with respiratory infections affecting 27.61% of the group, gastrointestinal disorders 20.71%, congenital heart disease 17.21%, and neurological conditions 13.77%. Additionally, ‘Other’ specified conditions were noted in 20.71% of the controls. The median NGAL level recorded was 79 ng/mL, serving as a benchmark for assessing renal function in non-CKD pediatric populations, enhancing the comparative understanding of the disease’s impact (Table 3).

Table 4 provides an in-depth analysis of proteinuria levels and CGA categories within a cohort of 54 pediatric CKD patients. Proteinuria was classified into three categories: A1 (normal to mildly increased) with 36 patients comprising 66.61%, A2 (moderately increased) with 14 patients at 25.99%, and A3 (severely increased) with four patients accounting for 7.41% of the group. Additionally, it outlined CGA categories ranging from CGA 1 to CGA 4, with eGFR ranging from ≥90 mL/min/m² to <30 mL/min/m². Median NGAL levels varied significantly across these categories, starting from 151 ng/mL in CGA 1 and rising to 951 ng/mL in CGA 4, with corresponding interquartile ranges indicating the variation in NGAL levels among the patients.

Table 5 details the laboratory findings for a study comparing pediatric patients with chronic kidney disease to a control group, encompassing 54 CKD patients and 29 controls. Key differences emerged in serum creatinine levels, with the CKD group displaying a significantly higher median of 148 µmol/L (IQR: 54.25, 636) compared to the control group’s 43 µmol/L (IQR: 36, 60), yielding a *p*-value of 0.0151. The estimated glomerular filtration rate (eGFR) similarly highlighted discrepancies, registering at 59.58 mL/min/m^2^ (SD ± 55.85) for the CKD group versus 126.10 mL/min/m^2^ (SD ± 26.34) for controls, with a significant *p*-value of 0.033. Conversely, calcium, phosphorus, and their product, along with alkaline phosphatase, serum protein, ferritin, hematocrit, and hemoglobin levels, showed no significant statistical difference.

In the subset of CKD patients undergoing continuous renal replacement therapy, marked clinical observations included a mean hemoglobin of 9.23 ± 1.72 g/dL and elevated ferritin levels with a median of 204.5 ng/mL (IQR: 84, 249.75 ng/mL). Serum neutrophil-gelatinase-associated lipocalin (NGAL) levels varied substantially between the study groups, with CKD patients showing a median of 453 ng/mL (IQR: 120.5, 854 ng/mL), starkly higher than the control’s 78 ng/mL (IQR: 76, 104 ng/mL). Notably, dialyzed patients presented the highest median NGAL levels at 1018.5 ng/mL (IQR: 825.5–1147), reflecting the severe nephrological impact in this subgroup (Table 5).

**Figure 2:**
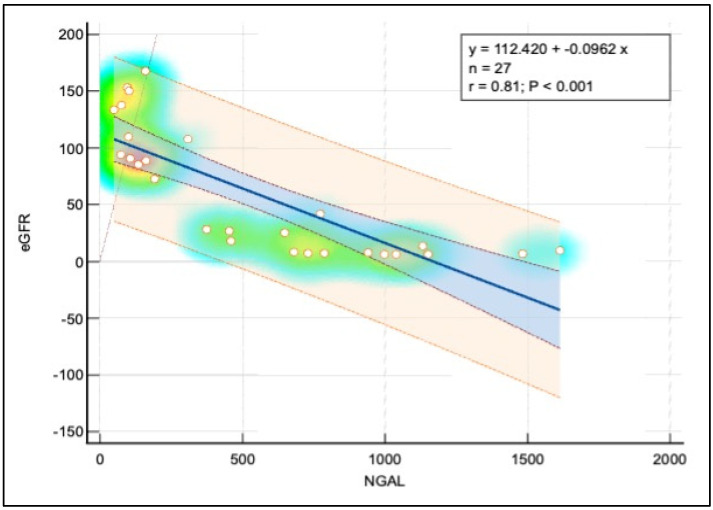
Correlation analysis between eGFR and NGAL.

Figure 2 illustrates a pronounced negative linear correlation between the estimated glomerular filtration rate and neutrophil-gelatinase-associated lipocalin. The scatter diagram enhanced with a regression line reveals this relationship, supported visually by a heat map and bounded by 95% confidence and prediction intervals in blue and orange, respectively. The heat map’s color gradient, ranging from red through yellow to blue, represents the density of data points, indicating clustering of observations. The most concentrated areas are marked in red, illustrating a high density of data points, which decreases from yellow to blue.

The accompanying analysis of variance (ANOVA) underscores the strength of this model, with a regression sum of squares at 52,586.08, explaining a substantial portion of the variance, and a residual sum of squares at 28,516.21, reflecting unexplained variance. The resulting F-ratio of 46.10 and an exceedingly low *p*-value (<0.0001) affirm the statistical significance of the regression model, accounting for approximately 64.84% of the variability in eGFR. However, it is crucial to recognize that correlation does not imply causation and external factors could influence these measures.

**Figure 3:**
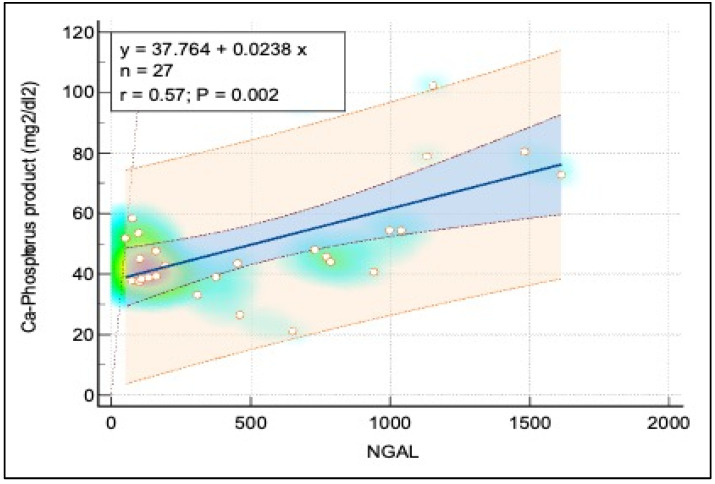
Correlation analysis between Ca-Phosphorus and NGAL.

**Figure 4:**
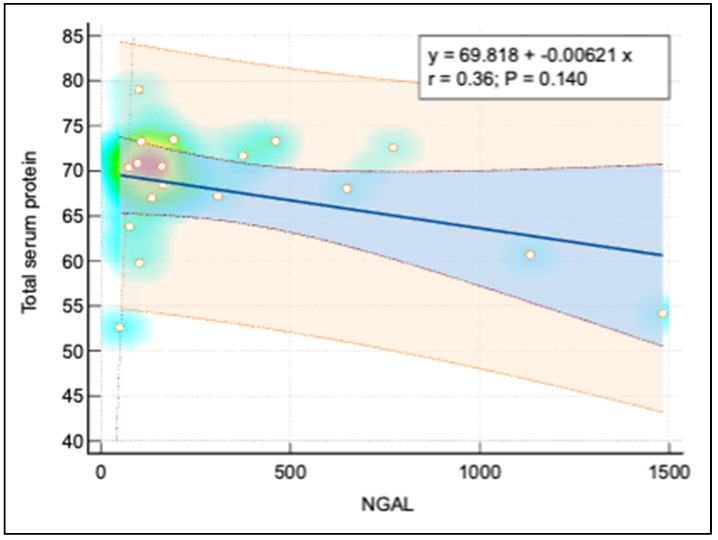
Correlation analysis between total serum protein and NGAL.

**Figure 5:**
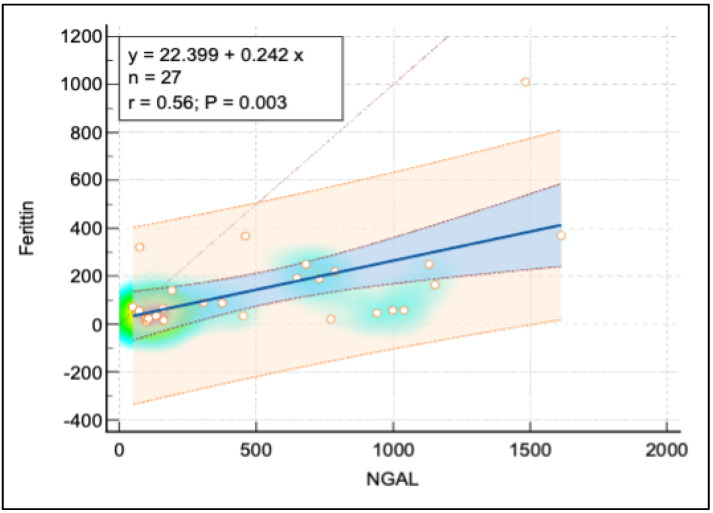
Correlation analysis between ferritin and NGAL.

Figure 3 and Figure 4 display further relationships: a moderate positive linear correlation between NGAL and both ferritin and the calcium-phosphorus (Ca*P) product, each depicted with a similar layout to Figure 5, using heat maps to signify point densities and show observational clustering. These correlations, like that in Figure 5, emphasize interconnected biomarkers in CKD, though they also caution against oversimplified causal interpretations without considering broader clinical contexts.

Patients on hemodialysis exhibit significantly higher median NGAL levels at 1023.7 ng/mL compared to 203.1 ng/mL in the non-dialysis group, indicating a substantial impact of dialysis on this biomarker, which is used to assess kidney injury and function. The interquartile ranges (148.7–254.3 ng/mL for no dialysis vs. 820.3–1142.8 ng/mL for hemodialysis) further emphasize the variability and elevated levels associated with more intensive treatment. Additionally, the estimated glomerular filtration rate (eGFR) and serum creatinine values reveal more severe renal impairment in the hemodialysis group (eGFR of 29.7 mL/min/m^2^ and creatinine of 297.8 µmol/L) compared to the no dialysis group (eGFR of 59.4 mL/min/m^2^ and creatinine of 119.6 µmol/L), as presented in Table 6.

There was a strong negative correlation between NGAL and eGFR (r = –0.81), suggesting that higher NGAL levels were associated with decreased renal function. NGAL and Ferritin showed a moderate positive correlation (r = 0.56), indicating that elevated NGAL levels might be linked to increased iron storage or altered iron metabolism. The correlation between NGAL and the Ca-Phosphorus Product was also moderately positive (r = 0.57), implying that higher NGAL levels were associated with disturbances in mineral metabolism, which is common in CKD. Additionally, a moderate negative correlation between NGAL and Total Serum Protein (r = –0.36) reflected the impact of CKD on nutritional status or protein loss. These correlations supported the potential role of NGAL as a biomarker for renal function decline and associated metabolic complications in pediatric CKD patients (Table 7).

Table 8 describes the results from linear regression analyses that examined the predictive value of NGAL on various dependent variables. NGAL was a significant predictor of eGFR (coefficient = –0.0972, *p* < 0.001), reinforcing the idea that as NGAL levels increased, renal function decreased. NGAL also significantly predicted ferritin levels (coefficient = 0.2453, *p* = 0.009), indicating a possible link between NGAL and iron metabolism. Moreover, NGAL significantly predicted the Ca-Phosphorus Product (coefficient = 0.0247, *p* = 0.006), suggesting its involvement in disorders of mineral metabolism. However, NGAL was not a significant predictor of Total Serum Protein (coefficient = –0.0067, *p* = 0.225), indicating that other factors might influence serum protein levels.

## 4. Discussion

### 4.1. Literature Findings and Critical Analysis

The study elucidates significant insights into the metabolic disruptions experienced by pediatric patients with chronic kidney disease, particularly through the lens of NGAL as a potential biomarker. One of the most compelling findings is the marked elevation of NGAL levels in patients undergoing continuous renal replacement therapy. This observation underscores NGAL’s potential role in reflecting the severity of renal dysfunction, with higher levels possibly indicating more severe kidney damage. The differentiation in NGAL levels between the general CKD cohort and those on CRRT provides a crucial indicator of the nephrological impact and the stress on kidney function in these young patients.

Another notable result involves the relationship between NGAL levels and the metabolic handling of calcium and phosphorus, as evidenced by the correlation with the calcium-phosphorus product. This relationship points to possible involvement of NGAL in the broader metabolic disturbances associated with CKD, including mineral metabolism, which is often a challenge in managing CKD due to its implications for bone health and cardiovascular stability. The study’s findings suggest that NGAL could be integral to the pathophysiology of these disturbances, marking a significant step forward in understanding the complex of mineral metabolism in bones and serum, in correlation with substances and diseases that impact kidney function [20,21,22,23,24,25].

Additionally, the study identifies the baseline characteristics and clinical profiles of the pediatric patients, revealing a majority in the mild to moderate stages of CKD, with a substantial fraction requiring CRRT. This demographic and clinical variability within the study cohort highlights the broad spectrum of CKD severity and its manifestations, which can vary significantly across pediatric patients. This variability necessitates a nuanced approach to treatment and management tailored to the individual patient’s clinical status and kidney function.

NGAL values were higher in the study group than in the control group. In the group of patients, the NGAL values were even higher. Different cut-off values for serum NGAL in CKD have been reported, one from a pediatric study being 190 ng/mL [26] and from an adult study 156 ng/mL [27].

Our results suggest a significant negative relationship between NGAL and eGFR. As eGFR tends to decrease, the serum NGAL increases. The model explains about 64.84% of the variability in eGFR. It must be considered that correlation does not imply causation, and other factors may also influence eGFR. Furthermore, it is also important to remember that these results are based on the specific sample of 27 observations, and results may vary with a different sample.

Our results are similar to those cited in the literature, where it has also been found that, in patients with CKD, NGAL concentrations were negatively correlated with the eGFR value, which reflected the severity of kidney damage [28,29,30].

It has been reported that observable damage in the distal convoluted tubule and Henle loop may be linked to elevated levels of NGAL [31], which may be a useful tool to assess CKD progression.

A study from 2023 reported a strong correlation between plasma NGAL concentrations and a higher chance of CKD with a new onset in the general population. In that specific group of population-based individuals, plasma NGAL does not appear to be any more useful than the evaluation of renal function in predicting the development of CKD in the future. Patients with certain kidney diseases, however, may experience this differently [32].

The relationship between neutrophil-gelatinase-associated lipocalin and iron metabolism indicators in chronic kidney disease patients has been increasingly noted. Research indicates inverse correlations between NGAL and several hematological parameters such as hemoglobin, hematocrit, serum iron, mean corpuscular volume (MCV), mean corpuscular hemoglobin (MCH), and transferrin saturation (TSAT) [9]. This suggests that as NGAL levels increase, markers of iron status tend to decrease, highlighting NGAL’s potential role in reflecting altered iron metabolism in CKD.

In our study sample, a moderate positive linear correlation was observed between ferritin and NGAL. This correlation could be attributed to the fact that patients in advanced stages of CKD, particularly those on erythropoietin-stimulating agents, typically show elevated ferritin levels, which correlate with increased NGAL. The median ferritin level in patients undergoing continuous renal replacement therapy was 204.5 ng/mL with an interquartile range from 84 to 249.75 ng/mL, aligning closely with values reported in existing literature [10]. Additionally, the median hemoglobin level of 9.23 g/dL in patients at CKD stage 5 is consistent with findings from other studies [10], further validating the study’s results.

While further research is essential, initial findings suggest that plasma NGAL could potentially serve as a more reliable indicator of iron status in CKD patients, particularly those requiring renal replacement therapy, compared to serum ferritin [33,34]. This could have significant implications for improving the management of iron deficiency and anemia in this patient population, enhancing overall treatment strategies.

Maintaining appropriate nutrition and linear growth in children with chronic kidney disease presents significant challenges due to multiple interrelated factors. Growth impairment in CKD can be attributed to nutritional deficiencies, metabolic acidosis, secondary hyperparathyroidism (SHPT), imbalances in the growth hormone/insulin-like growth factor 1 axis, pubertal dysfunction, appetite disorders, and the toxicity of certain medications such as corticosteroids [35]. This complex interplay highlights the critical balance required between ensuring adequate nutrient intake and avoiding complications that may impact cardiovascular health.

Cardiovascular risk factors are particularly prevalent in children and adolescents with CKD, with the most significant being obesity, arterial hypertension, altered glucose and lipid metabolism, and excess weight [36]. The cardiac complications that can arise from CKD and an improper diet include left ventricular hypertrophy, oxidative stress, and vascular calcifications [37]. In older patients, malnutrition and hypoalbuminemia are commonly observed. This demographic is at a heightened risk of malnutrition, defined by the loss of protein energy and micronutrient deficiencies [16].

Our findings suggest that neutrophil-gelatinase-associated lipocalin (NGAL) may play a role in the malnourishment and metabolic abnormalities of calcium and phosphorus seen in CKD. NGAL’s association with both the calcium-phosphorus (Ca*P) product and total serum proteins indicates its involvement in the broader metabolic disturbances affecting these patients. Given that poorly managed CKD is often linked to mineral bone disorders, the serum Ca*P ratio can serve as a predictor for future cardiovascular diseases. In adults with pre-dialysis CKD stages, conditions such as increased LDL cholesterol, oxidative stress, microinflammation, hypercholesterolemia, hypertriglyceridemia, and left ventricular hypertrophy have all been associated with higher Ca*P levels. Notably, the risk of cardiovascular disease over a 10-year period increases substantially when the Ca*P level exceeds 55 mg^2^/dL^2^ [37].

Based on our findings, it is important to incorporate alternative perspectives and findings from recent research. For instance, a study explored the utility of NGAL in the pediatric CKD population using a fuzzy logic approach due to inconsistent cut-off values and limited patient samples in prior research. This model aimed to enhance diagnostic accuracy and treatment decisions, highlighting a unique methodological approach in evaluating NGAL’s role in monitoring CKD progression [38].

Further, another study raised concerns about the effectiveness of NGAL as a reliable biomarker for CKD in children. It was noted that while NGAL is promising for detecting early signs of acute kidney injury, its role in chronic conditions, especially in varying stages of pediatric CKD, remains uncertain. This skepticism is supported by variability in NGAL levels influenced by factors such as infection or inflammation, which may not directly correlate with kidney function, thus challenging the biomarker’s utility in chronic scenarios [38,39]. These contrasting views present a necessary broader perspective on NGAL’s reliability and underscore the need for a more comprehensive assessment of its clinical utility in pediatric CKD management.

Cystatin C and kidney injury molecule-1 (KIM-1) are critical biomarkers that can complement NGAL in monitoring chronic kidney disease progression. Cystatin C is a protease inhibitor produced by all nucleated cells, considered more stable and reliable than creatinine for estimating glomerular filtration rate, as it is less affected by muscle mass, age, and sex. KIM-1, a type I cell membrane glycoprotein, is significantly upregulated in kidney cells in response to injury, making it a sensitive marker for both acute kidney injury and chronic renal damage [40]. These biomarkers could enhance diagnostic accuracy and help in the stratification of CKD severity, providing a broader view of renal health. However, our study did not incorporate these additional biomarkers due to financial constraints and their less frequent measurement in routine clinical settings, acknowledging this as a limitation and an area for future enhancement.

In our study, we noted the potential importance of correlating the slope of estimated glomerular filtration rate with neutrophil gelatinase-associated lipocalin levels, given that the eGFR slope is increasingly recognized as a vital indicator of chronic kidney disease progression. Although our data collection did not specifically target dynamic eGFR changes over time, we observed varying NGAL levels across different CGA categories, suggesting a possible link between eGFR and NGAL. This limitation underscores the need for future research explicitly designed to longitudinally measure both eGFR and NGAL, which could provide crucial insights into the mechanisms of CKD progression and help refine predictive models for this disease.

Looking ahead, there is significant potential to incorporate serum NGAL into a panel of biomarkers for assessing CKD progression in children. This approach could enhance our understanding of NGAL’s role and its applicability in a larger pediatric cohort. Moreover, NGAL could serve as a novel histological marker for patients requiring renal biopsy. The use of NGAL staining to assess the expression of this marker in tissue samples and its correlation with disease severity has already shown promise in adult studies, particularly in tracking the progression of IgA Nephropathy [38]. Such applications could provide critical insights into the pathophysiology of CKD and improve diagnostic and therapeutic strategies in pediatric nephrology. Additionally, future investigations should aim to explore the correlations between NGAL and secondary hyperparathyroidism in a pediatric setting. It is imperative that future studies include parathyroid hormone levels at the time of NGAL determinations, along with calcium and phosphorus levels, to provide a more complete picture of the metabolic disturbances associated with CKD.

### 4.2. Study Limitations

The study examining the correlation between NGAL levels and CKD progression, conducted with a cohort of 54 CKD patients and 29 controls from a single center in Timisoara, encountered several methodological challenges that raised questions about its findings. The diversity in CKD stages and severities within the cohort may have further compounded the inadequacy of the sample size for robust statistical analysis, particularly when attempting to discern associations amidst numerous potential confounding factors such as pharmaceutical regimens, dietary conditions, and genetic predispositions. The cross-sectional design of the study inherently limits its ability to establish causation, potentially undermining claims about NGAL’s predictive capabilities for CKD progression. Moreover, the specific demographic characteristics of the sample hinder the generalizability of the results. Given these limitations, the conclusions drawn about NGAL as a biomarker for monitoring CKD in clinical settings should be viewed as preliminary. Further research, ideally involving longitudinal studies with larger, more diverse populations, is essential to substantiate the utility of NGAL in predicting and managing the progression of CKD.

Our study’s methodology, primarily based on correlation coefficients and linear regression, may oversimplify the complex interactions between NGAL and kidney function in pediatric CKD. This approach neglects nonlinear correlations and variable interactions, potentially overstating the significance of findings due to unadjusted multiple comparisons, which is particularly problematic given our small sample size. Additionally, our regression models did not account for crucial variables such as age, treatment specifics, or CKD etiology, further limiting the reliability of our conclusions about NGAL as a biomarker.

## 5. Conclusions

Our study suggests a potential inverse relationship between the estimated glomerular filtration rate and neutrophil-gelatinase-associated lipocalin levels, which account for approximately 64.84% of the variability in eGFR. However, it is crucial to recognize that correlation does not equate to causation. The findings indicate that NGAL could be a useful clinical biomarker for CKD, but they also highlight the need for cautious interpretation due to the study’s limitations, such as the lack of adjustments for multiple comparisons and control for confounding variables. Future studies involving larger, multicentric cohorts are necessary to establish more definitive reference and cut-off values for NGAL and to confirm its utility in a clinical setting. Additionally, considering the complex nature of CKD, a combination of multiple biomarkers would likely enhance the accuracy of monitoring disease progression. Effective management of pediatric CKD also requires a holistic approach that includes not only pharmacological treatments but also nutritional support and comprehensive care coordination.

## Figures and Tables

**Table 1:** The baseline characteristics of the study population.

Characteristic	Sample Size (*n* = 54)
Male gender	28 (51.85%)
Urban areas	34 (62.86%)
Age at the moment of examination (months)	173; (92.5, 192)
Weight at the moment of examination (kg)	37.3; (20.5, 55.7)
Height (cm)	135.33 ± 28.75

**Table 2:** The causes of CKD in the present cohort.

Causes of CKD	Sample Size (*n* = 54)
Kidney anomalies	
Renal dysplasia	6 (11.11%)
Collecting system anomalies	
Ureteropelvic junction stenosis	4 (7.41%)
Ureterocel	2 (3.7%)
Bladder anomalies	
Vesicoureteral reflux—primary	2 (3.7%)
Urethral anomalies	
Posterior urethral valve	8 (14.81%)
SDNS/SRNS	4 (7.41%)
Chronic Glomerulonephritis	
IgA Nephropathy	2 (3.7%)
GSFS	2 (3.7%)
ANCA-MPO + vasculitis	4 (7.41%)
Neurogenic bladder	10 (18.52%)
aHUS	4 (7.41%)
Nephronophthisis	4 (7.41%)
Status post typical HUS	2 (3.7%)

**Table 3:** Control group characteristics.

Characteristic/Disease	Number of Controls (*n* = 29)	Percentage (%)
Age (months)	122; (61, 179)	
Gender		
- Male	16	55.19%
- Female	13	44.81%
Residence		
- Urban	18	62.01%
- Rural	11	37.91%
Underlying Diseases		
- Respiratory Infections	8	27.61%
- Gastrointestinal Disorders	6	20.71%
- Congenital Heart Disease	5	17.21%
- Neurological Conditions	4	13.77%
- Other (Specify)	6	20.71%
Median NGAL (ng/mL)	79.3	

**Table 4:** Distribution of proteinuria levels and CGA categories in pediatric CKD patients.

Proteinuria Category	Number of Patients (*n* = 54)	Percentage (%)	CGA Category	eGFR Range (mL/min/m²)	Median NGAL (ng/mL)	Range NGAL (ng/mL)
A1 (Normal to mildly increased)	36	66.61%	CGA 1	≥90	151	131–201
A2 (Moderately increased)	14	25.99%	CGA 2	60–89	302	251–353
A3 (Severely increased)	4	7.41%	CGA 3	30–59	603	551–653
			CGA 4	<30	951	901–1001
Total	54	100%				

**Table 5:** The main laboratory findings.

Laboratory Assessment	Sample Size *n* = 54	Control Group *n* = 29	*p*-Value
Serum creatinine (μmol/L)	148; (54.25, 636)	43; (36, 60)	0.0151
eGFR (mL/min/m^2^)	59.58 ± 55.85	126.10 ± 26.34	0.033
Ca (mmol/L)	2.39; (2.15, 2.45)	2.26; (2.21, 2.42)	0.5464
Phosphorus(mmol/L)	1.67; (1.32, 2.05)	1.52; (1.48, 1.58)	0.5555
Ca-Phosphorus (mg^2^/dL^2^)	45.14; (38.93, 54.39)	43.41; (42.01, 44.58)	0.5473
ALP (mmol/L)	204.38 ± 114.08	163.44 ± 84.81	0.3890
Serum Protein (g/L)	67.60 ± 6.96	67.23 ± 4.58	0.8716
Serum ferritin (ng/mL)	72; (34.25, 213.25)	64; (46.25, 115.25)	0.7561
Hematocrit (%)	33.60; (28.80; 37.65)	35.30; (32.10, 38.17)	0.5466
Hemoglobin (g/dL)	11.40; (9.55, 13)	12.40; (10.90, 13.05)	0.3476

**Table 6:** Impact of hemodialysis on key clinical markers in pediatric chronic kidney disease patients.

Treatment Type	Median NGAL (ng/mL)	Interquartile Range NGAL (ng/mL)	eGFR (mL/min/m^2^)	Median Serum Creatinine (µmol/L)
No Dialysis	203.1	148.7–254.3	59.4	119.6
Hemodialysis	1023.7	820.3–1142.8	29.7	297.8

**Table 7:** Correlation matrix.

	NGAL	eGFR	Ferritin	Ca-Phosphorus	Total Serum Protein
NGAL	1	–0.81	0.56	0.57	−0.36
eGFR	−0.81	1	−0.45	−0.48	0.32
Ferritin	0.56	−0.45	1	0.59	−0.28
Ca-Phosphorus	0.57	−0.48	0.59	1	−0.41
Total Serum Protein	−0.36	0.32	−0.28	−0.41	1

**Table 8:** Regression analysis table.

Dependent Variable	Independent Variable	Coefficient	Standard Error	*t*-Statistic	*p*-Value
eGFR	NGAL	−0.0972	0.0154	−6.32	<0.001
Ferritin	NGAL	0.2453	0.0879	2.79	0.009
Ca-Phosphorus	NGAL	0.0247	0.0083	2.98	0.006
Total Serum Protein	NGAL	−0.0067	0.0054	−1.24	0.225

## Data Availability

The information is contained within this article in its entirety. For additional information, please feel free to inquire with either the original author or the corresponding author. The public’s access to the data is restricted as a result of the patient privacy standards that regulate the handling of clinical data.
